# Arsenic on the Hands of Children: Wang et al. Respond

**Published:** 2005-06

**Authors:** Zhongwen Wang, Elena Kwon, Hongquan Zhang, Gian S. Jhangri, Xiufen Lu, Xing-Fang Li, X. Chris Le, Nelson Fok, Stephan Gabos

**Affiliations:** Department of Public Health Sciences, University of Alberta, Edmonton, Alberta, Canada, E-mail: xc.le@ualberta.ca; Environmental Health, Capital Health, Edmonton, Alberta, Canada; Health Surveillance Branch, Alberta Health and Wellness, Edmonton, Alberta, Canada

In our study of arsenic on children’s hands (Kwon et al. 2004), we measured arsenic in water samples in which participating children washed both hands after playing on selected playgrounds. The hand-washing water was filtered, and the soluble arsenic concentration in the filtrate was determined by inductively coupled plasma mass spectrometry. In response to Kissel’s comment that we did not measure insoluble arsenic, we analyzed the arsenic levels in the insoluble residue collected on the filter and summarized the unpublished data here. Results from the analysis of 64 samples from the CCA playgrounds and another 63 samples from the non-CCA playgrounds are available upon request. The total amount of arsenic in the insoluble residue collected in the hand-washing water of 64 children from the eight CCA playgrounds was 418 ± 601 ng (mean ±SD), compared to 172 ± 278 ng in the hand-washing water of 63 children from the eight non-CCA playgrounds. The total arsenic collected in the hand-washing water (insoluble arsenic on the filter plus water-soluble arsenic in the filtrate) was 934 ± 940 ng for the CCA playground and 265 ± 311 ng for the non-CCA playgrounds. The maximum amount of total arsenic collected from children’s hands was 4,743 ng (4.7 μg). This is compared with the 3.9 μg that we reported previously (Kwon et al. 2004).

To provide a perspective of relative contribution of this amount of arsenic to the overall exposure to arsenic, in our article (Kwon et al. 2004) we included references for the average daily dietary ingestion of total arsenic:

38 μg (15 μg for children 1–4 years of age) for Canada (Dabeka et al. 1993), 62 μg for the United States (Gartrell et al. [1985]), 89 μg for the United Kingdom (Food Additives and Contaminants Committee 1984), 55 μg for New Zealand (Dick et al. 1978), and 160–280 μg for Japan (Tsuda et al. 1995). A range of arsenic species that have different toxicities may be present in food (Le et al. 2004). Estimated daily dietary intake of inorganic arsenic was 8.3–14 μg in the United States (Yost et al. 1998), 4.8–12.7 μg in Canada (Yost et al. 1998), and 15–211 μg in Taiwan (Schoof et al. 1998).

We did not monitor children’s hand-to-mouth activity because this behavior has already been documented in the literature (Reed et al. 1999; Tulve et al. 2002). Our intent was to provide direct measurements of the amount of arsenic on children’s hands. We recognize the importance of these other studies, as we pointed out in our “Conclusions” (Kwon et al. 2004):

The results—along with other information, such as the frequency and habit of hand-to-mouth activity, efficiency of transfer of arsenic from hands to mouth, and repeated contact of hands with CCA-treated wood surface after hand-to-mouth activity—are useful for assessing children’s exposure to arsenic.

We have measured arsenic in sequential hand-washings and found that most arsenic was present in the first hand-washing (unpublished data). Results of arsenic in hand-washings of three children before and after playing on a CCA playground are available upon request. The amount of arsenic in the second washing was < 10% of that in the first washing, suggesting that the arsenic on children’s hands is readily washed off with water. Therefore, we conclude that children should “wash their hands after playing to reduce their potential exposure to arsenic” (Kwon et al. 2004).

Biomonitoring of arsenic species in urine samples from children who play on CCA-treated structures and children who do not could be useful if the ingestion of arsenic from dietary sources would not be a major confounder.
